# Illumination Effect on Bipolar Switching Properties of Gd:SiO_2_ RRAM Devices Using Transparent Indium Tin Oxide Electrode

**DOI:** 10.1186/s11671-016-1431-8

**Published:** 2016-04-27

**Authors:** Kai-Huang Chen, Kuan-Chang Chang, Ting-Chang Chang, Tsung-Ming Tsai, Shu-Ping Liang, Tai-Fa Young, Yong-En Syu, Simon M. Sze

**Affiliations:** Department of Electrical Engineering and Computer Science, Tung Fang Design Institute, Kaohsiung, Taiwan; Department of Materials and Optoelectronic Science, National Sun Yat-Sen University, Kaohsiung, Taiwan; Department of Physics, National Sun Yat-Sen University, Kaohsiung, Taiwan; Advanced Optoelectronics Technology Center, National Cheng Kung University, Tainan, Taiwan; Department of Mechanical and Electro-Mechanical Engineering, National Sun Yat-Sen University, Kaohsiung, Taiwan; Department of Electronics Engineering and Institute of Electronics, National Chiao Tung University, Hsinchu, Taiwan

**Keywords:** Nonvolatile memory, Illumination effect, Gadolinium, Silicon oxide, RRAM

## Abstract

To discuss the optoelectronic effect on resistive random access memory (RRAM) devices, the bipolar switching properties and electron-hole pair generation behavior in the transparent indium tin oxide (ITO) electrode of Gd:SiO_2_ thin films under the ultraviolet (*λ* = 400 nm) and red-light (*λ* = 770 nm) illumination for high resistance state (HRS)/low resistance state (LRS) was observed and investigated. In dark environment, the Gd:SiO_2_ RRAM devices exhibited the ohmic conduction mechanism for LRS, exhibited the Schottky emission conduction and Poole-Frankel conduction mechanism for HRS. For light illumination effect, the operation current of the Gd:SiO_2_ RRAM devices for HRS/LRS was slightly increased. Finally, the electron-hole pair transport mechanism, switching conduction diagram, and energy band of the RRAM devices will be clearly demonstrated and explained.

## Background

Magnetic random access memory (MRAM), ferroelectric random access memory (FeRAM), and phrase change memory (PCM) devices are indispensable to various nonvolatile electronic applications in portable electron devices [1–4]. Because of the excellent compatibility integrated circuit (IC) processes, long retention cycles, low operation voltage, and low electric consumption, the various resistive random access memory (RRAM) devices are investigated and discussed in recent memory device search [5–10]. Among these RRAM device applications, the different metal element-doped silicon dioxide thin films prepared by various physical vapor disposition methods are widely considered and fabricated [1–10].

According to previous studies, the bipolar resistance switching and initial metallic filament forming properties of the various structure RRAM devices using indium tin oxide (ITO) electrode for the high resistance state (HRS) and low resistance state (LRS) are investigated for experimental details [5–12]. Besides, the illumination effect induced the electron-hole pair generation in switching operation current of the RRAM devices for the transparent ITO electrode is not widely discussed.

In this study, the ITO/Gd:SiO_2_/TiN structure of the RRAM devices was prepared by gadolinium-doped SiO_2_ layer between of titanium nitride (TiN) and ITO electrode. In addition, the bipolar switching resistive properties of Gd:SiO_2_ RRAM devices for HRS/LRS affected by the ultraviolet (*λ* = 400 nm) and red-light (*λ* = 770 nm) illumination effect were also discussed later.

## Methods

**Fig. 1:**
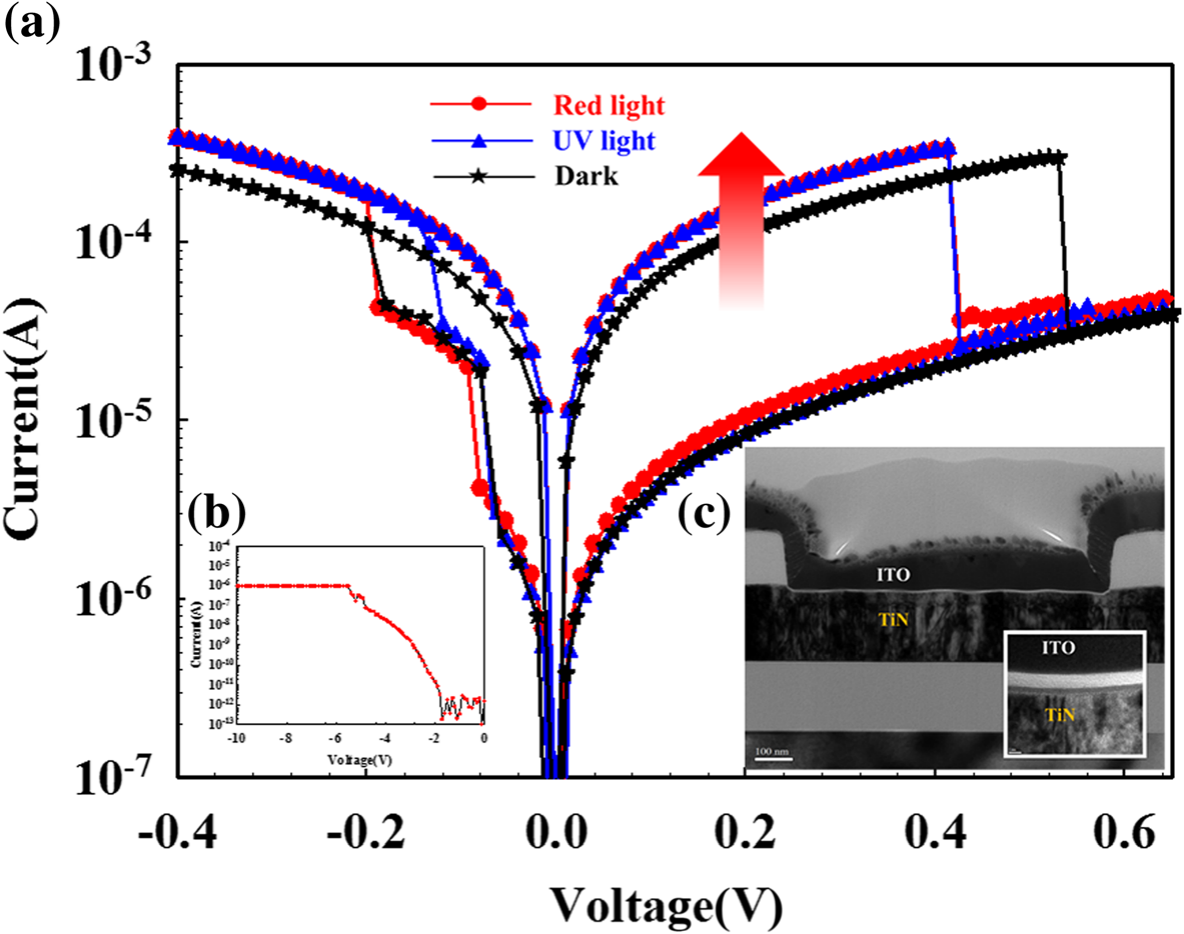
(**a**) The typical bipolar behavior of the Gd:SiO_2_ thin film RRAM devices for (**b**) initial electrical forming process and (**c**) using the metal-insulator-metal (MIM) structure. (blue lines: *λ* = 770nm, red lines: *λ* = 400nm, black lines: standard)

The metal-insulator-metal (MIM) structure samples were fabricated and investigated to the bipolar switching properties of RRAM devices by co-sputtering technology with pure silicon dioxide and gadolinium targets in Fig. 1(c). The Gd:SiO_2_ thin film was about 10 nm of thickness. In addition, the sputtering power was the rf power of 200 W and dc power of 10 W for silicon dioxide and gadolinium targets, respectively. To form ITO/Gd:SiO_2_/TiN structure, the ITO top electrode with a thickness of 200 nm was also deposited on Gd:SiO_2_ thin film by rf sputtering. The typical switching resistance properties of Gd:SiO_2_ RRAM devices are obtained by Agilent B1500 semiconductor parameter analyzer. To discuss the illumination effect on Gd:SiO_*x*_ RRAM devices, the switching conduction diagram for electron-hole pair carrier transport properties is measured and described by the ultraviolet (*λ* = 400 nm) and red-light (*λ* = 770 nm) environment.

## Results and Discussion

In Fig. 1(a), the typical *I*-*V* switching curves of the Gd:SiO_2_ thin film RRAM device was exhibited the bipolar switching behavior properties. After the initial electrical forming process in Fig. 1(b), the LRS/HRS states of the Gd:SiO_2_ RRAM device was reached and observed. To define reset process, the operation switching current of the devices was gradually decreased from LRS to HRS by sweeping the positive bias over the reset voltage. To avoid the failure and broken situation of RRAM devices, the compliance current was limited to 1 μA. For inverted bipolar switching resistive behaviors, the transmission electron in metallic filament path early captured by the lots of oxygen vacancy in ITO top electrode of Gd:SiO_2_ RRAM devices was proved and investigated in Fig. 1(a) [12].

**Fig. 2:**
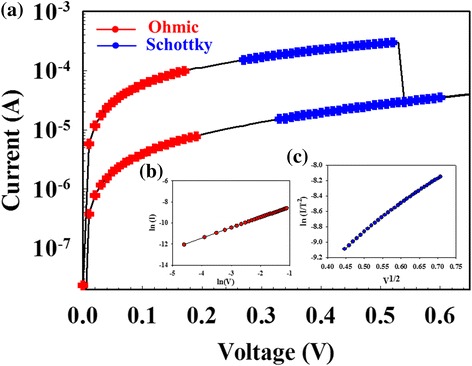
(**a**) The *I*-*V* switching properties of Gd:SiO_*x*_ RRAM devices in dark environment for (**b**) ohmic conduction and (**c**) Schottky emission mechanism

**Fig. 3:**
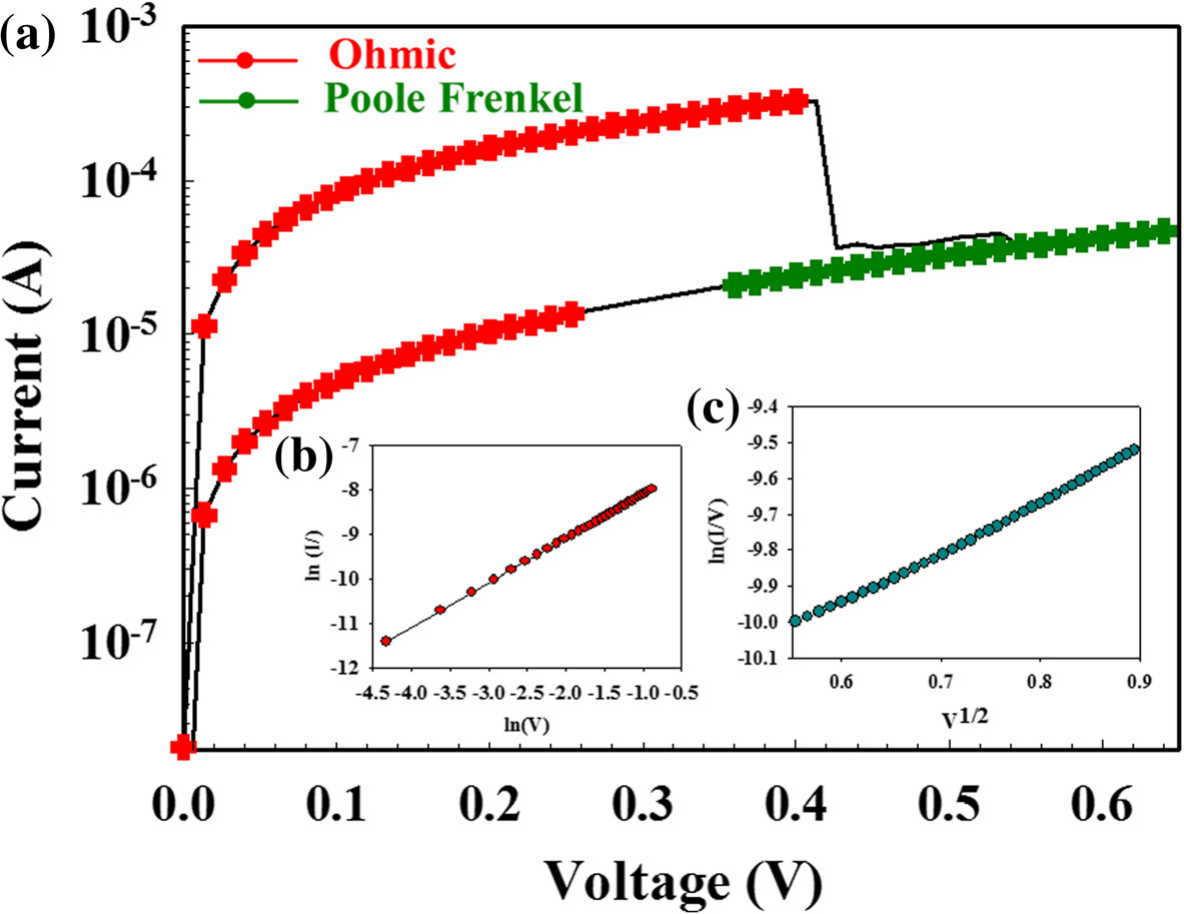
(**a**) The *I*-*V* switching properties of Gd:SiO_*x*_ RRAM devices in illumination environment for (**b**) ohmic conduction and (**c**) Poole-Frankel emission mechanism

To investigate the optoelectronic effect on the ITO electrode of Gd:SiO_*x*_ RRAM devices, the bipolar switching properties measured by ultraviolet-light (*λ* = 400 nm) and red-light (*λ* = 770 nm) illumination environment was shown in Figs. 2 and 3. In set state, all switching operation current of RRAM devices for LRS/HRS were slightly increased and induced by light illumination effect. In dark environment, the *I*-*V* curves of the RRAM devices exhibited the ohmic conduction for low voltage and exhibited Schottky emission mechanism for high voltage in Fig. 2(a). In light environment, the operation current of RRAM devices for LRS/HRS was exhibited to ohmic conduction mechanism in Fig. 3(a). The Schottky emission mechanism for HRS was observed for high applied voltage.

To describe the physical mechanism for optoelectronic effect on ITO electrode of the RRAM devices, the electron-hole pair carrier generated in conduction mechanism and electron transport path diagram was explained in Figs. 2 and 3. In Fig. 2(a), the RRAM device for HRS was transferred from the Schottky emission mechanism to Poole-Frankel mechanism in illumination effect environment [9–11]. In Fig. 2(b, c), the electrons of initial metallic filament path in the Gd:SiO_2_ thin film RRAM devices jumped from the defect activation energy, induced the leakage current, and exhibited the Poole-Frankel mechanism in illumination environment.

In Fig. 3(a), the RRAM device for LRS was transferred from the Schottky emission mechanism to ohmic conduction mechanism in illumination environment. In Fig. 3(b), the RRAM devices exhibited the Schottky emission conduction for high applied voltage. The barrier height of oval-shaped depletion region in ITO thin films was formed by the oxygen-rich atoms surrounding tip metallic filament. In Fig. 3(c), the ohmic conduction mechanism was caused by lots of intrinsic carrier generation of electron transport behavior in metallic filament of Gd:SiO_2_ thin films.

**Fig. 4:**
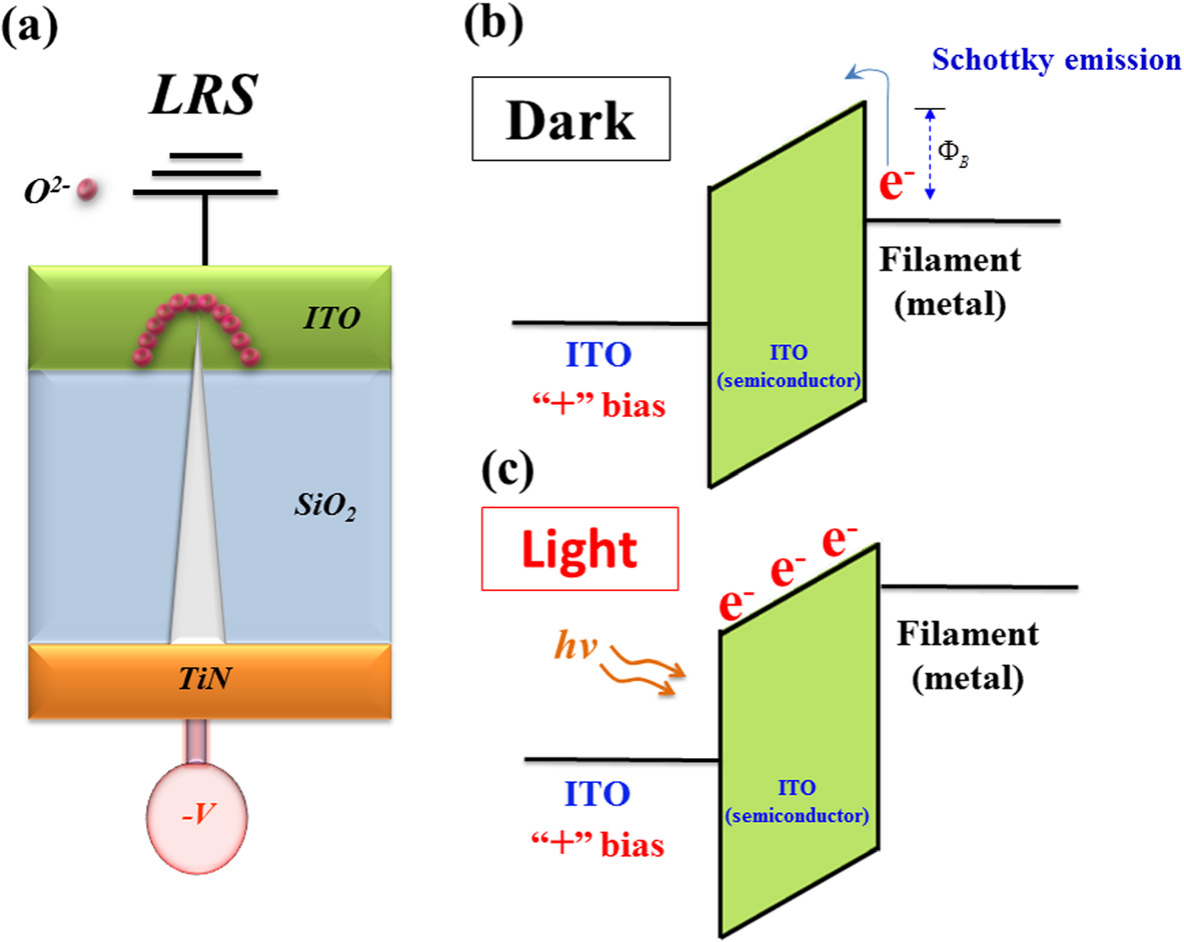
**a** Initial metallic filament model, **b** energy band model in dark, and **c** energy band model in light of the Gd:SiO_*x*_ RRAM devices for LRS

**Fig. 5:**
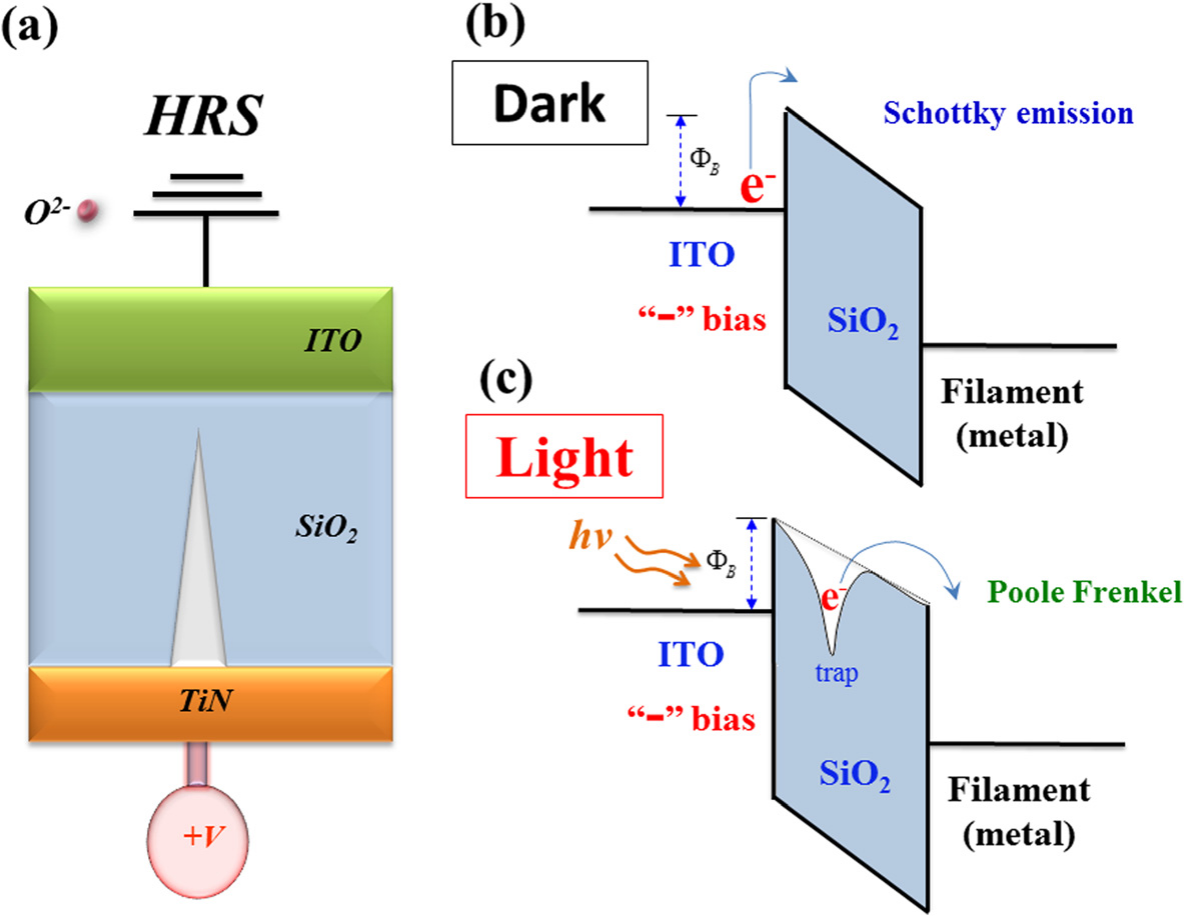
**a** Initial metallic filament model, **b** energy band model in dark, and **c** energy band model in light of the Gd:SiO_*x*_ RRAM devices for HRS

To further discuss and prove the above inference detail for optoelectronic effect, the energy band model of physical conduction mechanism was drawled and described in Figs. 4 and 5. In Fig. 4a, the oval-shaped depletion region formed by the oxygen ions in ITO electrode of the Gd:SiO_2_ thin film RRAM devices for LRS was gradually accumulated. Then, the metallic path tip was passed through the oval-shaped depletion region in ITO electrode for continuing applied high negative voltage. Besides, the semiconducting ITO thin films exhibit the n-type semiconductor for energy band diagram. In Fig. 4b, the transmission electron in metallic filament path transferred and overcome the barrier height was exhibited the schottky conduction mechanism for continuing applied voltage. In illumination environment, the electron-hole pair of ITO electrode was generated in conduction/valance band and exhibited the ohmic conduction mechanism in Fig. 4c.

In HRS, the oxygen ions return the TiN electrode and recombined the metallic filament tip in Gd:SiO_2_ thin films for high positive applied voltage in Fig. 5a. In Fig. 5b, the transmission electron of ITO electrode overcome the barrier height in Gd:SiO_2_ thin film region which was also found for the Schottky conduction mechanism. For continuing positive applied voltage, the electron was departed from the trap and exhibited the Poole-Frankel conduction mechanism in Fig. 5c.

## Conclusions

For the ultraviolet (*λ* = 400 nm) and red-light (*λ* = 770 nm) illumination environment, the bipolar switching properties and conduction mechanism of Gd:SiO_2_ RRAM devices using transparent ITO electrode for HRS/LRS states were measured and investigated. Besides, the switching operation current for LRS/HRS was slightly increased by ultraviolet and red-light illumination effect. For the Schottky emission mechanism transferred to the Poole-Frankel mechanism in illumination environment for HRS, the leakage current of RRAM devices was caused by electron jump from the defect activation energy. For illumination environment effect in LRS, the Schottky emission mechanism transferred to ohmic conduction of the RRAM devices induced by lots of electron-hole pair generation was proved.
